# Fluvoxamine, an anti-depressant, inhibits human glioblastoma invasion by disrupting actin polymerization

**DOI:** 10.1038/srep23372

**Published:** 2016-03-18

**Authors:** Keiichiro Hayashi, Hiroyuki Michiue, Hiroshi Yamada, Katsuyoshi Takata, Hiroki Nakayama, Fan-Yan Wei, Atsushi Fujimura, Hiroshi Tazawa, Akira Asai, Naohisa Ogo, Hiroyuki Miyachi, Tei-ichi Nishiki, Kazuhito Tomizawa, Kohji Takei, Hideki Matsui

**Affiliations:** 1 Department of Physiology, Okayama University Graduate School of Medicine, Dentistry and Pharmaceutical Sciences, Okayama, Japan; 2 Department of Pharmacy, Okayama University Hospital, Okayama, Japan; 3 Department of Neuroscience, Okayama University Graduate School of Medicine, Dentistry and Pharmaceutical Sciences, Okayama, Japan; 4 Department of Pathology, Okayama University Graduate School of Medicine, Dentistry and Pharmaceutical Sciences, Okayama, Japan; 5 Department of Molecular Physiology, Faculty of Life Sciences, Kumamoto University, Kumamoto, Japan; 6 Center for Innovative Clinical Medicine, Okayama University Hospital, Okayama, Japan; 7 Department of Gastroenterological Surgery, Okayama University Graduate School of Medicine, Dentistry and Pharmaceutical Sciences, Okayama, Japan; 8 Center for Drug Discovery, Graduate School of Pharmaceutical Sciences, University of Shizuoka, Shizuoka, Japan; 9 Division of Pharmaceutical Sciences, Okayama University, Graduate School of Medicine, Dentistry and Pharmaceutical Sciences, Okayama, Japan

## Abstract

Glioblastoma multiforme (GBM) is the most common malignant brain tumor with a median survival time about one year. Invasion of GBM cells into normal brain is the major cause of poor prognosis and requires dynamic reorganization of the actin cytoskeleton, which includes lamellipodial protrusions, focal adhesions, and stress fibers at the leading edge of GBM. Therefore, we hypothesized that inhibitors of actin polymerization can suppress GBM migration and invasion. First, we adopted a drug repositioning system for screening with a pyrene-actin-based actin polymerization assay and identified fluvoxamine, a clinically used antidepressant. Fluvoxamine, selective serotonin reuptake inhibitor, was a potent inhibitor of actin polymerization and confirmed as drug penetration through the blood–brain barrier (BBB) and accumulation of whole brain including brain tumor with no drug toxicity. Fluvoxamine inhibited serum-induced ruffle formation, cell migration, and invasion of human GBM and glioma stem cells *in vitro* by suppressing both FAK and Akt/mammalian target of rapamycin signaling. Daily treatment of athymic mice bearing human glioma-initiating cells with fluvoxamine blocked tumor cell invasion and prolonged the survival with almost same dose of anti-depressant effect. In conclusion, fluvoxamine is a promising anti-invasive treatment against GBM with reliable approach.

Glioblastoma multiforme (GBM) is the most common malignant primary brain tumor, with a median survival of approximately 1 year. Despite advances in diagnostics and treatment, the prognosis for GBM has not significantly improved in recent decades^1^. This poor prognosis is mainly due to the highly invasive nature of GBM cells. Diffused GBM cell invasion into surrounding normal brain tissue prevents complete surgical resection of GBM tumors and results in recurrence. Furthermore, in the central nervous system, most anti-cancer drugs, including molecular-targeted drugs, forming a first line of treatment against various cancers are ineffective because the BBB prevents their delivery into the brain^2^. Therefore, the development of novel anti-invasive drugs that can permeate the BBB is essential for treatment of GBM.

Recent studies have identified CD133^+^ glioma-initiating cells (GICs) that exhibit stem cell-like properties^3^,^4^.These GICs possess capacities for tumorigenesis, self-renewal, and differentiation into multiple cell types, such as neurons, astrocytes, and oligodendrocytes^4^,^5^. GICs have been shown to be highly invasive and resistant to chemotherapy and radiotherapy^6^,^7^,^8^. Therefore, GICs are thought to be responsible for the poor prognosis of GBM and constitute a potential target for GBM therapy.

Tumor cell migration and invasion require dynamic reorganization of the actin cytoskeleton^9^,^10^. Migrating cells produce membrane protrusions, such as filopodia, lamellipodia, invadopodia, focal adhesions, and stress fibers^11^. Because these structures of migrating cells require precise regulation of actin polymerization and depolymerization, control of actin polymerization in tumor cells on the leading edge of the tumor may inhibit invasion and migration of GBM cells into normal brain.

In terms of drug development and clinical applications, the cost of development and unexpected side effects just before clinical use obstruct the process from basic research to clinical use. As a result, finding new uses for existing clinically used drugs, termed drug repositioning or repurposing, is an alternative strategy for drug discovery and development^12^. This approach has been widely attempted and has been successful in some cases (e.g., aspirin as an anti-platelet medication, sildenafil for erectile dysfunction, etc.)^12^,^13^. Because the pharmacokinetics of most existing clinically used drugs have already been studied, the effective dose, possible side effects, cost are already known and the time required to bring these drugs to market can be reduced^14^.

## Results

### Fluvoxamine found to inhibit actin polymerization using a new screening method for quantitative determination of actin polymerization

**Figure 1:**
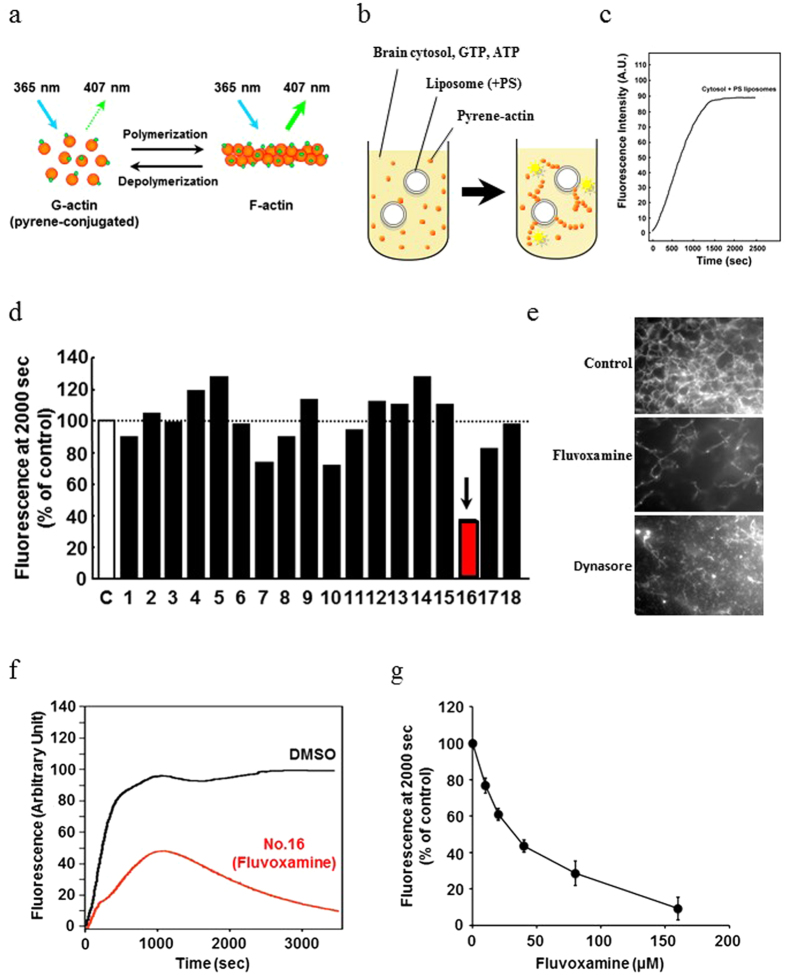
Pyrene-actin-based screening identified fluvoxamine as a potent inhibitor of actin polymerization. (**a**–**c**) Schematic diagram of screening performed. Pyrene-labeled G-actin was polymerized by stimulation with liposomes (50% phosphatidylcholine, 50% phosphatidylserine) in the reaction buffer containing mouse brain cytosol, ATP, and GTP. Ex/Em: 365/407 nm. (**d**) Drug No. 16 (fluvoxamine, arrow) effectively inhibited actin polymerization. Each drug (40 μM) was added to the reaction mixture to assess its effect on actin polymerization. The fluorescence intensity of each sample was normalized to the DMSO control at 2000 s. (**e**) Actin polymerization was monitored by a visual assay. Mouse brain cytosol pretreated with 80 μM fluvoxamine (middle panel) or 80 μM dynasore (bottom panel) for 30 min before incubation. Control cytosol was pretreated with 0.1% DMSO (upper panel). Scale bar, 10 mm. (**f**) Representative time course of actin polymerization in the presence of DMSO (black line) or 40 μM fluvoxamine (red line). (**g**) Fluvoxamine inhibited actin polymerization in a concentration-dependent manner, and its IC_50_ was ~30 μM. Values are mean ± SEM from three experiments.

Reorganization of the actin cytoskeleton is essential for cancer cell migration and invasion. Therefore, we established a new drug screening method for *in vitro* quantitative determination of actin polymerization and screened clinically used drugs that can penetrate the BBB. To test the new screening method, we first screened inhibitors of actin polymerization from among 18 clinically used drugs that can permeate the BBB (Table 1) using a pyrene-actin-based actin polymerization assay. This assay is based on enhancement of the fluorescence of pyrene-labeled G-actin (monomer) that occurs during polymerization (Fig. 1a–c). Each drug was added to the reaction mixture at a concentration of 40 μM, and the fluorescence of pyrene-actin was measured. We found that drug No. 16, the antidepressant fluvoxamine, exhibited the most potent inhibition against actin polymerization (Fig. 1d).

Using an *in vitro* actin assembly assay, we directly observed F-actin formation and depolymerization by TIRF microscopy. Compared with the control, fluvoxamine and positive control (dynasore) samples clearly exhibited relatively low formation of F-actin. The inhibitory effect of fluvoxamine on actin polymerization was concentration dependent, and its IC_50_ was approximately 30 μM (Fig. 1g). On the other hand, fluvoxamine did not inhibit actin polymerization when pyrene-actin (Fig. 1e) was polymerized in the absence of mouse brain cytosol (Fig. S1A) and when pyrene-actin and Arp2/3 were co-incubated (Fig. S1B). These results suggest that fluvoxamine inhibits actin polymerization by inhibiting actin polymerization-related proteins in brain cytosol but not by directly inhibiting actin or Arp2/3.

### Fluvoxamine suppresses serum-induced lamellipodia formation in GBM cell lines

**Figure 2:**
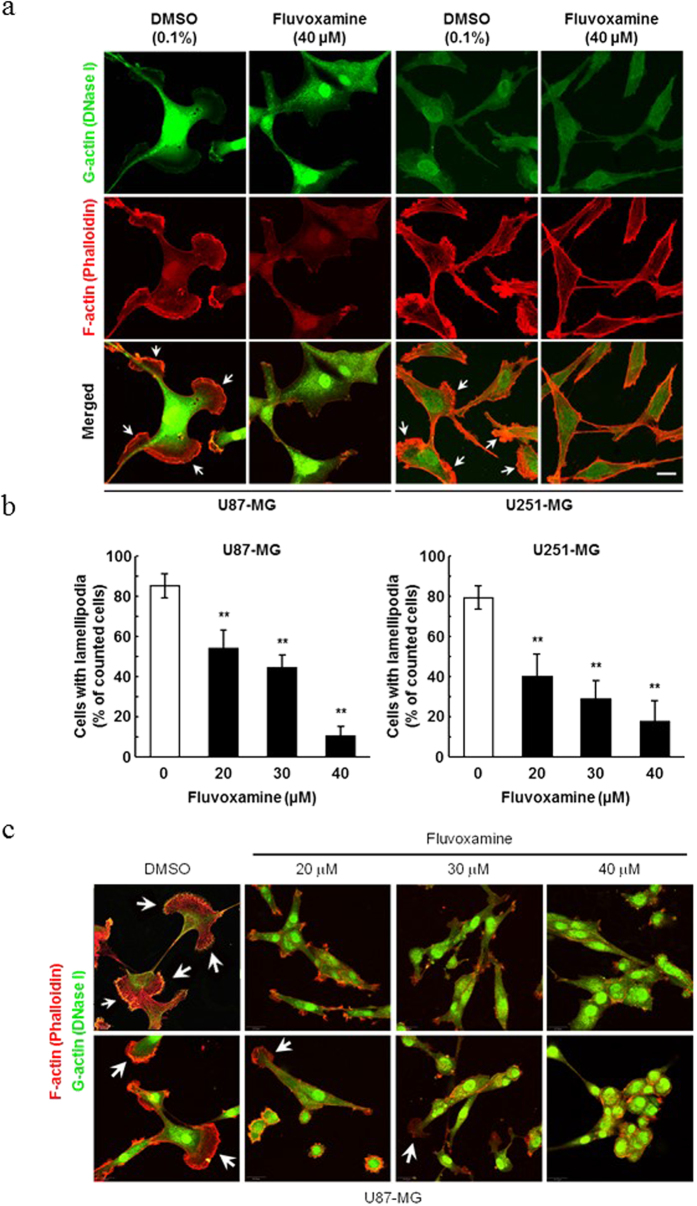
Fluvoxamine suppressed serum-induced lamellipodia formation. (**a**) Human GBM cell lines (U87-MG and U251-MG) were serum-starved for 24 h, treated with either vehicle (0.1% DMSO) or fluvoxamine (40 μM) for 15 min, and stimulated with 10% FBS for 15 min. G-actin (green) and F-actin (red) were stained with Alexa Fluor 488-conjugated DNase I and Alexa Fluor 555-conjugated phalloidin, respectively. Arrows indicate lamellipodia. Scale bar, 20 μm. (**b**) Fluvoxamine inhibited serum-induced lamellipodia formation in a dose-dependent manner. Values are mean ± SEM from 5–14 independent fields. **p < 0.01 compared with vehicle, non-repeated measures ANOVA with Dunnett’s *post-hoc* test. (**c**) U87 glioma ruffle formation was inhibited in a dose-dependent manner by fluvoxamine and observed by confocal microscopy with G-actin (green) and F-actin staining (red), as above (**a**).

To investigate whether fluvoxamine suppresses the formation of lamellipodia, which are important for cell migration, serum-starved human GBM cell lines (U87-MG and U251-MG) were treated with 40 μM fluvoxamine or vehicle (0.1% DMSO) and then stimulated with FBS to induce lamellipodia formation. Serum stimulation induced significant lamellipodia formation in vehicle-treated cells but not in fluvoxamine-treated cells (Fig. 2a). Fluvoxamine induced the inhibition of ruffle formation in a dose-dependent manner in U87-MG. In addition, the ratio of G-actin in tumors was markedly higher than that in the DMSO control (Fig. 2c). Fluvoxamine effectively blocked serum-induced lamellipodia formation in a dose-dependent manner (Fig. 2b).

### Fluvoxamine inhibits GBM cell migration and invasion *in vitro*

**Figure 3:**
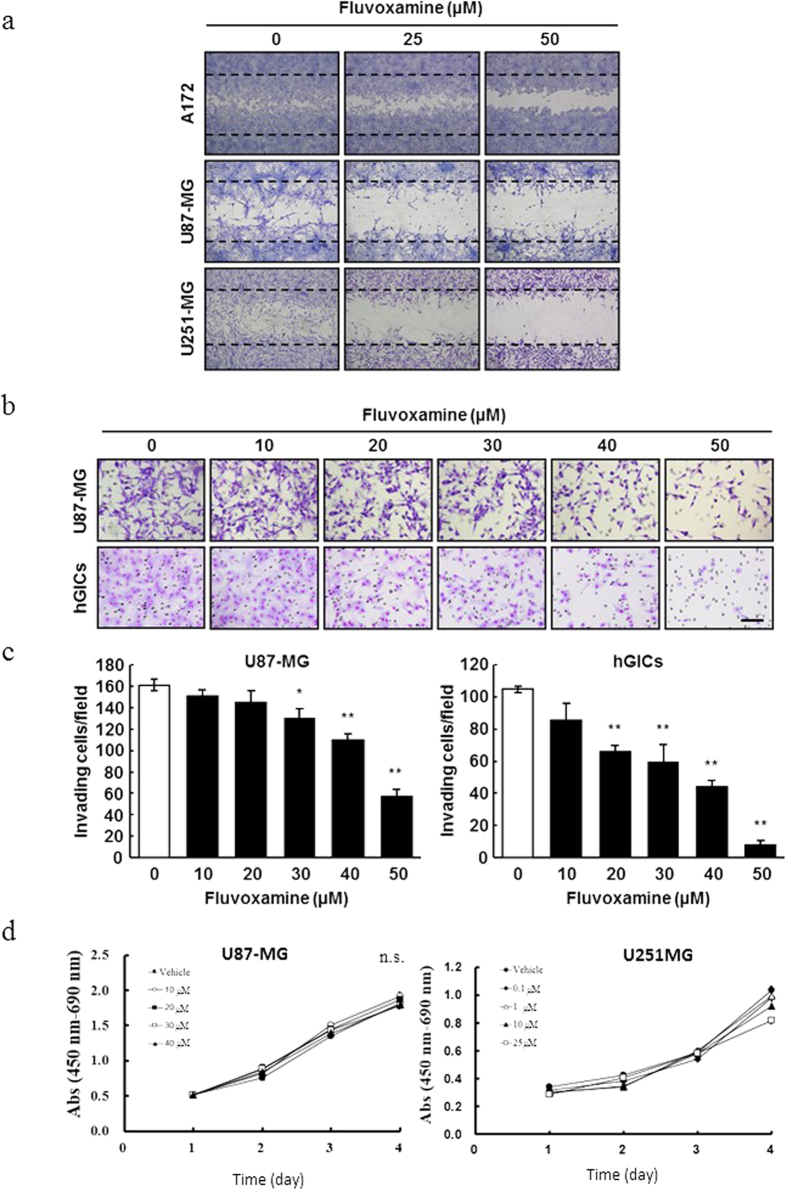
Fluvoxamine inhibited human GBM cell and GIC migration and invasion *in vitro*. (**a**) Fluvoxamine decreased GBM cell migration in a dose-dependent manner. A172, U87-MG, and U251-MG cells were serum-starved for 24 h, treated with fluvoxamine (0, 25, or 50 μM), and processed for wound-healing assay. (**b,c**) Fluvoxamine inhibited GBM cell invasion in a dose-dependent manner. U87-MG and hGICs were treated with various doses of fluvoxamine and processed for Matrigel invasion assay. Scale bar, 100 μm. Values are mean ± SEM from three independent experiments. *p < 0.05, **p < 0.01 compared with vehicle, non-repeated measures ANOVA with Dunnett’s *post-hoc* test. (**d**) Fluvoxamine did not have no effect to cell proliferation, U87MG and U251MG with WST-1 assay for four days. Values are mean ± SEM from these independent experiments, and statistical analysis was performed using the Student’s T-test by comparing fluvoxamine administrated groups (U87-MG: 10, 20, 30, or 40 μM, U251MG: 0.1, 1, 10, or 25 μM) versus non-treated (vehicle) group . These data showed no significant among these groups in glioma cell proliferation.

To examine the *in vitro* effects of fluvoxamine on GBM cell migration and invasion, we performed a wound-healing and Matrigel invasion assay. In the wound-healing assay, fluvoxamine effectively inhibited the migration of three different human GBM cell lines (A172, U87-MG, and U251-MG) in a dose-dependent manner (Fig. 3a). Furthermore, this drug also inhibited invasion of U87-MG and hGICs in a dose-dependent manner (Fig. 3b). To examine the effect of fluvoxamine on glioma cell proliferation, we did WST-1 assay with the different dose of fluvoxamine (Fig. 3d). These data showed that fluvoxamine had no effect against these two cell lines, U87MG and U251MG. This indicated that fluvoxamine directly behaved to inhibited invasion, but not cell proliferation.

### Daily administration of fluvoxamine inhibits GBM cell invasion and prolongs survival in mice bearing GBM tumors

**Figure 4:**
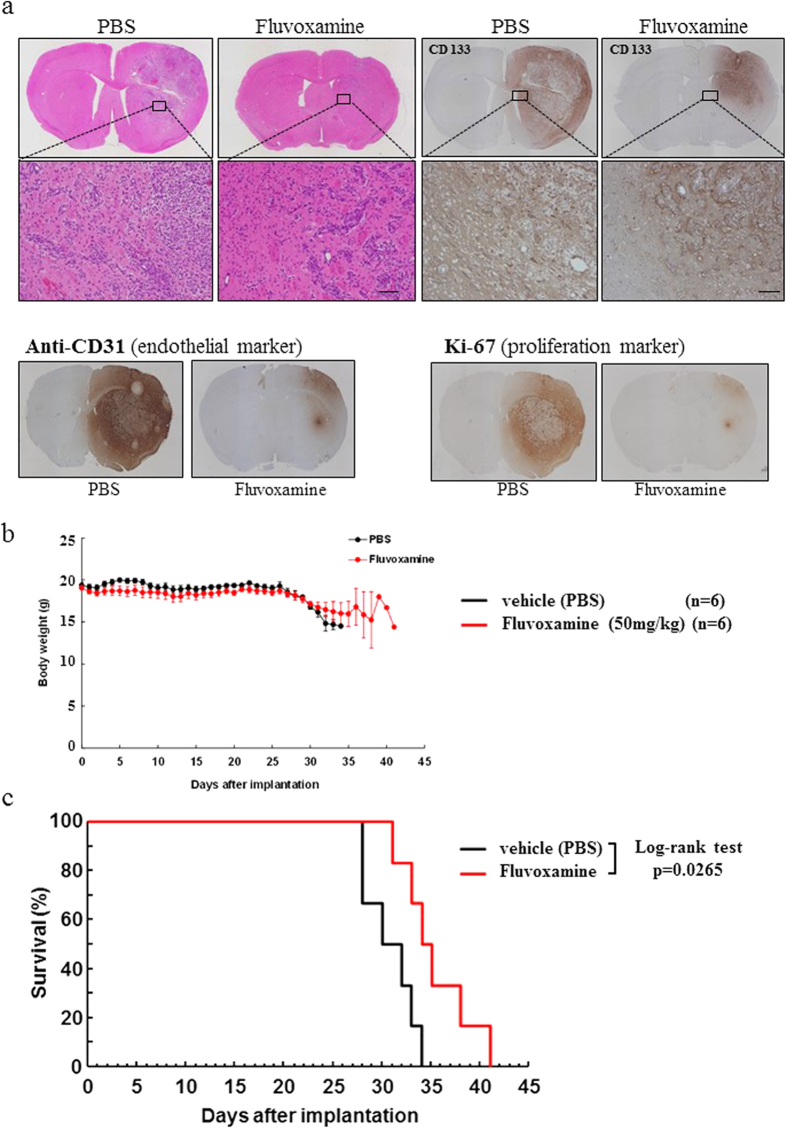
Fluvoxamine inhibited invasion of hGICs *in vivo* and prolonged the survival of GBM mice. (**a**) Daily treatment with fluvoxamine inhibited tumor cell invasion *in vivo*. hGICs (10^3^ cells) were implanted into the right striatum of nude mice and the treatment was initiated 1 week after implantation. Fluvoxamine maleate (50 mg/kg/day) or vehicle (PBS) was injected intraperitoneally every day. Representative images of H&E staining of brain sections from mice 4 weeks after tumor implantation. (**b**) Daily *i.p*. injection of fluvoxamine prolonged survival of mice bearing intracranial hGICs (10^4^ cells). Kaplan–Meier plot showing survival of mice treated with vehicle (PBS, black line) or fluvoxamine (red line). Scale bar, 100 μm. n = 6 each group; *p < 0.05, log-rank test.

To assess the *in vivo* anti-invasive effect of fluvoxamine, we implanted hGICs into the right striatum of nude mice. One week after implantation, daily treatment with fluvoxamine maleate (50 mg/kg/day, intraperitoneally once a day) was initiated. This dose of fluvoxamine was shown to be effective in the forced-swimming test, which is an experimental model of depression in rodents^15^,^16^. Our results showed that daily fluvoxamine had no effect on mouse body weight, suggesting that this dosage does not cause serious side effects (Fig. 4b).

Four weeks after tumor cell implantation, we performed histological analysis of tumors using hematoxylin and eosin (H&E) staining and immunohistochemistry (IHC) with several specific antibodies. We observed a huge tumor in the right hemisphere where hGICs were implanted in mice, and highly invasive tumor cells were found in the surrounding normal brain of mice treated with vehicle (PBS) by H&E staining (Fig. 4a). The PBS-treated control sample showed that glioma and hGICs invaded normal brain at the tumor border in a disorderly fashion (Fig. 4a). On the other hand, tumors of mice treated with fluvoxamine were significantly smaller compared to control (Fig. 4a). In addition, invasive tumor cells formed island-like shapes, exhibited colonizing morphology, and aggregated together at the tumor border.

IHC with CD133 was used to show glioma stem cell distribution, while CD31 acted as a vascular endothelial proliferation marker and Ki-67 was used as a cell proliferation marker. CD133^+^ cells were found localized to a small site corresponding to the tumor location with fluvoxamine treatment. In contrast, CD133^+^ cells in the PBS control group spread to the area surrounding the tumor, as shown by H&E staining. CD31 staining revealed that the entire tumor area was occupied by CD31^+^ cells in the control sample, whereas very few CD31^+^ cells were observed in the fluvoxamine-treated group. Ki67 IHC showed that highly proliferative cells covered almost the entire tumor area apart from the tumor center in the PBS group; alternatively, a very small number of Ki67^+^ cells were seen in the fluvoxamine group. Furthermore, the survival of mice treated with fluvoxamine was significantly longer than that of PBS-treated mice (Fig. 4c). These results suggest that an effectively anti-depressive dose of fluvoxamine is sufficient to inhibit hGICs invasion.

### Fluvoxamine inhibits both FAK and Akt/mammalian target of rapamycin (mTOR) signaling pathways

**Figure 5:**
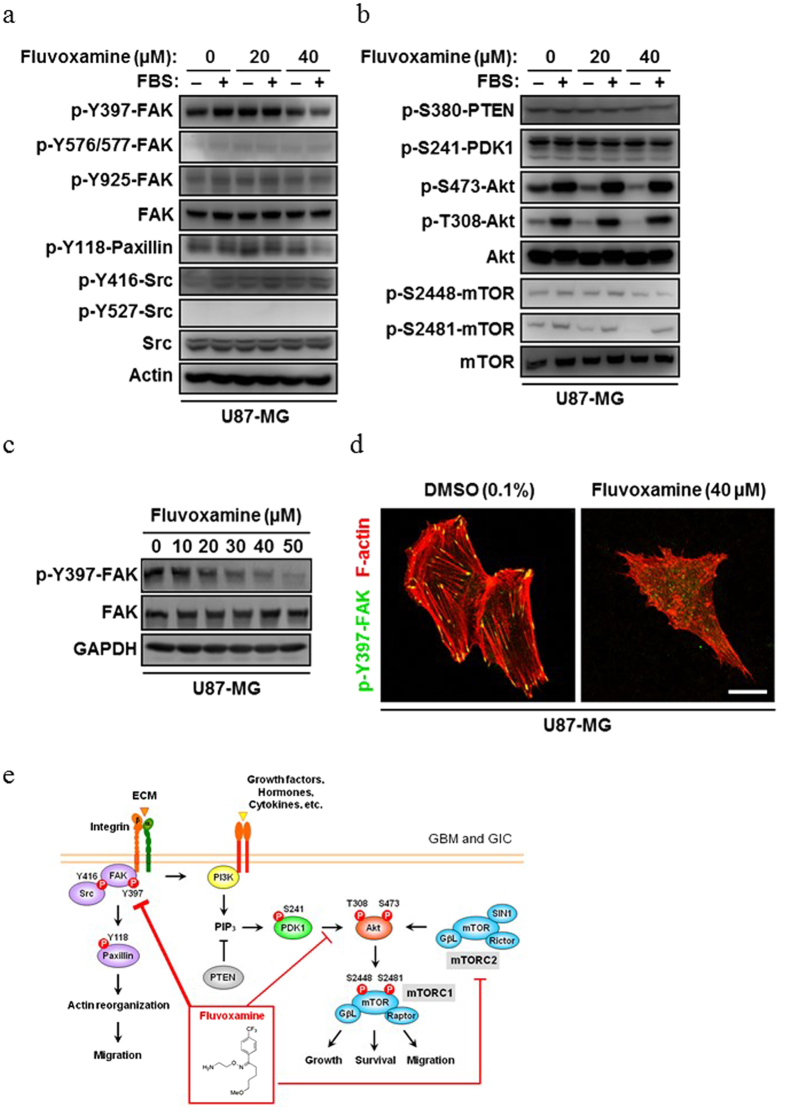
Mechanism of glioma invasion inhibition by fluvoxamine with the suppression of FAK and Akt/mTOR signaling. (**a,b**) Fluvoxamine decreased the phosphorylation of FAK (Y397), paxillin (Y118), Akt (S473 and T308), and mTOR (S2448 and S2481) in U87-MG cells. Serum-starved U87-MG cells were treated with fluvoxamine (0, 20, or 40 μM) for 15 min and then stimulated with or without 10% FBS for 15 min. Equal amounts of protein from each sample were subjected to Western blot. (**c**) Fluvoxamine decreased FAK autophosphorylation in a dose-dependent manner. U87-MG cells were treated with various doses of fluvoxamine for 1 h and processed for Western blot. (**d**) Fluvoxamine suppressed FAK autophosphorylation and disrupted stress fibers and focal adhesions. U87-MG cells were treated with vehicle (0.1% DMSO) or fluvoxamine (40 μM) for 30 min, fixed, and stained with anti-p-Y397-FAK (green) and Alexa Fluor 555-conjugated phalloidin (red). Scale bar, 20 μm. (**e**) A schema of the complete mechanism of inhibition of actin polymerization and the tumor suppressor effect against GBM cells and hGICs by fluvoxamine.

To elucidate the molecular mechanism behind the anti-invasive effect of fluvoxamine, we examined various signaling pathways that are involved in actin polymerization. Serum-starved U87-MG cells were pretreated with fluvoxamine (0, 20, or 40 μM) for 15 min, stimulated with FBS for 15 min, collected, and the level of each protein was analyzed by Western blot. Fluvoxamine decreased the level of FAK phosphorylation at an activating autophosphorylation site (Y397; Fig. 5a) and decreased phosphorylation of Akt at T308 and S473 (Fig. 5b). However, there were no significant changes in the activities of PTEN and phosphoinositide-dependent kinase 1 (PDK1; Fig. 5b), which are upstream of Akt in the phosphoinositide-3-kinase (PI3K)/Akt pathway. Fluvoxamine also suppressed phosphorylation of mTOR at S2448 and S2481 (Fig. 5b), which are phosphorylated by Akt^17^,^18^. The phosphorylation levels of other actin reorganization-related proteins, ezrin/radixin/moesin, vasodilator-stimulated phosphoprotein, and cofilin, were unchanged by fluvoxamine treatment (Fig. S2).

U87-MG cells were treated with fluvoxamine at various doses for 1 h and FAK phosphorylation at Y397 were analyzed. Fluvoxamine treatment suppressed phosphorylation of Y397-FAK in a dose-dependent manner (Fig. 5c). Immunofluorescent staining of p-Y397-FAK in U87-MG cells revealed that p-Y397-FAK signals completely stopped in fluvoxamine-treated cells, together with disruption of stress fibers and focal adhesions compared to vehicle-treated cells (Fig. 5d). We also found that fluvoxamine decreased paxillin phosphorylation at Y118 (Fig. 5b), which is a direct substrate of FAK (Fig. 5e)^19^. These results suggest that fluvoxamine may exert anti-invasive activity by targeting FAK and Akt/mTOR signaling (Fig. 5e).

## Discussion

The development of new anti-cancer agents is expensive and time consuming. Generally, pharmaceutical companies use large-scale gene analysis, *in silico* analysis, and high-throughput screening for the identification of new drugs against new cancer targets; however, these techniques may require a large developmental budget and long-term development. In addition, once new cancer drugs reach the clinical field, unexpected side effects sometimes occur and, in some cases, the drugs must be withdrawn. Alternatively, when the use of a new cancer drug is expanded to malignant brain tumors, especially GBM, their distribution, pharmacokinetics, and BBB permeability become an issue.

The concept of drug repositioning has recently drawn attention and involves old clinical drugs being put to practical use for another disease and another target. In the present study, we report identification of the antidepressant fluvoxamine as a potent inhibitor of actin polymerization, which is essential for cancer cell migration and invasion. Consistent with this, fluvoxamine effectively inhibited the formation of lamellipodia, focal adhesions, and stress fibers, as well as the migration and invasion of human GBM cells *in vitro*. Fluvoxamine also suppressed FAK and Akt/mTOR signaling. Furthermore, daily administration of fluvoxamine to mice bearing hGICs blocked tumor cell invasion and prolonged the mouse survival period. Taken together with the fact that fluvoxamine has been safely used for a long time for the treatments of various mental disorders, our findings suggest that fluvoxamine is a promising candidate for anti-invasion therapy for GBM.

Specifically, we identified fluvoxamine, a clinically used antidepressant, as a potent anti-invasive drug for the treatment of GBM. Here we demonstrated that fluvoxamine effectively inhibits actin polymerization, which is essential for cancer cell migration and invasion. Consistent with this, fluvoxamine-treated GBM cells failed to form lamellipodia, focal adhesions, and stress fibers and displayed decreased migration and invasion *in vitro*. We also demonstrated that fluvoxamine suppressed FAK and Akt/mTOR signaling pathways, suggesting that inhibition of these signaling pathways results in disrupted actin polymerization. Furthermore, daily administration of fluvoxamine to mice bearing hGICs blocked tumor cell invasion and prolonged the survival of these mice. These results suggest that fluvoxamine may be a promising anti-invasive drug against GBM and that a strategy targeting the actin cytoskeleton has the potential to inhibit tumor cell motility and overcome the poor prognosis of GBM.

Although fluvoxamine was observed to inhibit actin polymerization *in vitro* (Fig. 1), it did not involve a direct effect on actin (Fig. S1). Moreover, fluvoxamine decreased autophosphorylation of FAK at Y397 (Fig. 5a, 5c) and disrupted focal adhesions and stress fibers (Fig. 5d). FAK has been shown to be overexpressed in various types of cancer, including GBM^20^,^21^,^22^, and to modulate actin polymerization and lamellipodial protrusion^23^, suggesting that FAK is a potential target for anti-invasive therapies for various cancers. In fact, Y15, a small-molecule inhibitor of FAK autophosphorylation, was shown to decrease the invasivity of human GBM cell lines^24^. Consequently, it is possible that the inhibition of actin polymerization by fluvoxamine is mediated via FAK signaling. However, the autophosphorylation state of FAK at Y397 is cell attachment-dependent^25^. Thus, we cannot rule out the possibility that decreased FAK autophosphorylation in fluvoxamine-treated GBM cells was caused by cell detachment.

We also observed that fluvoxamine decreased the phosphorylation of Akt at S473 and T308 (Fig. 5a). Akt-S473 is phosphorylated by mTOR complex 2 (mTORC2) and DNA-activated protein kinase^26^,^27^,^28^. Because mTORC2 has been shown to regulate actin polymerization^29^, it is possible that fluvoxamine directly and/or indirectly affects mTORC2 activity. Although T308 of Akt was previously shown to be phosphorylated by PDK1 through the PI3K pathway^30^,^31^, the activity of PDK1 (p-S241-PDK1) was not affected by FBS stimulation (Fig. 5a), suggesting that Akt-T308 is phosphorylated by unknown protein kinases in U87-MG cells, and fluvoxamine inhibits this activity. Unlike protein-protein interactions, it is difficult to examine direct interactions between small molecules and proteins. In addition, there are more than 100 proteins involved in the regulation of actin dynamics^32^. Thus, the molecular mechanism by which fluvoxamine inhibits actin polymerization has not yet been elucidated and further understanding of the precise mechanism requires additional studies.

We show that the effective dose of fluvoxamine required to inhibit lamellipodia formation and migration and invasion of GBM cells *in vitro* was approximately 20–30 μM (Figs 2 and 3). A clinical study using fluorine magnetic resonance spectroscopy demonstrated that the steady-state brain concentration of fluvoxamine exceeded 20 μM in some patients administered a clinical dose (100–300 mg/day) of fluvoxamine for the treatment of depression^33^. Accordingly, our study demonstrates that the clinically anti-depressive dose of fluvoxamine is sufficient to prevent invasion of GBM cells. Consistent with this, we observed that the anti-depressive dose of fluvoxamine significantly blocks tumor invasion and prolongs the survival of GBM-bearing mice without obvious side effects (Fig. 4). Fluvoxamine is selectively incorporated into the central nervous system and its brain concentration is 10- to 20-fold higher than its plasma concentration^33^,^34^, suggesting it can inhibit GBM invasion without severe peripheral side effects. Furthermore, it is reported that selective serotonin reuptake inhibitors, including fluvoxamine, can be safely used for the treatment of depression in patients with GBM^35^. Taken together, these findings demonstrate that fluvoxamine is a promising candidate for safe and effective GBM therapy.

Most anti-cancer drugs currently used are anti-proliferative, affecting cell division or DNA synthesis, whereas very few drugs effectively inhibit tumor cell invasion^36^. A recent study reported that imipramine-blue (IB), a derivative of the tricyclic anti-depressant imipramine, inhibits the invasion of GBM cells *in vitro*, and liposome-encapsulated IB prolongs the survival of tumor-bearing rats when combined with liposomal doxorubicin^37^. These findings suggest that a combination of standard therapies with anti-invasive drugs could be a useful approach for the treatment of GBM.

Finding new uses for existing clinically used drugs, termed drug repositioning or repurposing, is an alternative strategy for drug discovery and development^12^. This approach has been widely attempted and has been successful in some cases, such as the use of aspirin as an anti-platelet medication and sildenafil in erectile dysfunction^12^,^13^. Because the pharmacokinetics of most existing clinically used drugs have already been studied, the effective dose and possible side effects are well known, the cost and time required to bring these drugs to market can be reduced^14^. Given that fluvoxamine has been used safely for various mental disorders, it is a potential candidate for drug repositioning.

In conclusion, we demonstrated that a clinically used anti-depressant, fluvoxamine, effectively inhibits actin polymerization and blocks the invasion of GBM cells into normal brain tissues. Our findings and concepts suggest the therapeutic potential of fluvoxamine as an anti-invasive drug in the treatment of GBM and provide evidence that targeting actin dynamics is a novel therapeutic approach for the treatment of GBM.

## Materials and Methods

### Antibodies and reagents

The primary antibodies used are described in the Supplementary Methods (Doc. S1). Drugs used in this study are listed in Table 1. For *in vitro* experiments, drugs were dissolved in dimethylsulfoxide (DMSO). For animal experiments, fluvoxamine maleate was dissolved in phosphate-buffered saline (PBS) and then filter-sterilized using a polyvinylidene membrane filter (0.22-μm pore size, Millipore).

### Actin polymerization assay

Quantitative analysis of actin polymerization was performed as previously described^38^. Briefly, pyrene-conjugated actin (0.3 mg/ml) was incubated for 10 min with mouse brain cytosol (12 mg/ml), an ATP generating system (1 mM ATP, 8 mM creatine phosphate, 8 U/ml phosphocreatine kinase), and 0.1 mM GTP in assay buffer (20 mM HEPES-KOH [pH 7.4], 100 mM KCl, 1 mM MgCl_2_, 0.1 mM EDTA, 1 mM dithiothreitol). Liposomes (120 μM; 50% phosphatidylcholine, 50% phosphatidylserine) were added to the mixture, and pyrene fluorescence was measured using a spectrophotometer (F-2500, Hitachi) at an Ex/Em: 365/407 nm. To assess the inhibitory effects of the tested drugs, each drug (40 μM) was added to the reaction mixture before stimulation with liposomes. The fluorescence intensity of each sample at 2000 s was normalized to 100% of the vehicle (DMSO) control.

### *In vitro* actin assembly assay

For a visual assay of actin assembly, mouse brain cytosol (9 mg/ml) was supplemented with 76 mM liposomes composed of 50% phosphatidylserine, 50% phosphatidylcholine, and rhodamine-actin (0.01 mg/ml) (Molecular Probes, CA) in the presence of the ATP-generating system, 1.3 mM MgCl_2_, and 0.1 mM EGTA. The mixture was incubated for 30–60 min and examined by TIRF microscopy (Olympus, Tokyo, Japan). Metamorph software was used for operating the microscope and image processing.

### Cell lines and culture

Human GBM cell lines U87-MG, U251-MG, and A172, and human glioma-initiating cells (hGICs)^39^ were used. Details are described in the Supplementary Methods (Doc. S1).

### Lamellipodial formation and immunocytochemistry

Cells were cultured on Matrigel (growth factor-reduced, BD Biosciences)-coated coverslips and serum-starved in low-serum (0.1% fetal bovine serum [FBS]) Dulbecco’s Modified Eagle’s Medium (DMEM) for 24 h. The cells were treated with either fluvoxamine or vehicle (0.1% DMSO) for 15 min and then stimulated with 10% FBS for 15 min to induce lamellipodia formation. The cells were washed with PBS, fixed with 4% paraformaldehyde for 10 min, and blocked with PBS containing 5% normal goat serum (Abcam) and 0.3% Triton X-100 for 30 min. G-actin and F-actin were stained with Alexa Fluor 488-conjugated DNase I (Life Technologies) and Alexa Fluor 555-conjugated phalloidin (Life Technologies), respectively.

For immunocytochemistry, cells were incubated with an anti-p-Y397-FAK antibody (44-624G, Life Technologies) for 1 h then incubated with Alexa Fluor 488-conjugated goat anti-rabbit IgG (Life Technologies) for 1 h. Coverslips were mounted with 50% glycerol, and confocal images were acquired using a confocal laser microscope (FV-300, Olympus).

### Wound-healing assay

The wound-healing assay was performed as previously described^40^. Cells were cultured on Matrigel-coated dishes until confluence and serum-starved in low-serum DMEM for 24 h. Monolayer wounds were produced by scratching with a 200-μl pipette tip. The medium was replaced with low-serum DMEM containing either fluvoxamine or vehicle (0.1% DMSO). The cells were allowed to migrate into the wounded area for 24 h, fixed with methanol, and stained with 5% Giemsa solution.

### Matrigel invasion assay

The *in vitro* invasion assay was performed using a BioCoat Matrigel invasion chamber (24-well format, BD Biosciences) according to the manufacturer’s instructions. In brief, 2 × 10^5^ cells were seeded in low-serum DMEM in the upper chamber. The lower chamber was filled with DMEM containing 10% FBS as a chemoattractant. Fluvoxamine or vehicle (0.1% DMSO) was added to both upper and lower chambers. After a 24-h incubation, non-invading cells in the upper chamber were removed with a sterile cotton swab. The filters of inserts were fixed with methanol and stained with 5% Giemsa solution. The number of invading cells on the lower surface of the filter was counted.

### Western blotting

Western blotting was performed as described in the Supplementary Methods (Doc. S1) and all of data in original form at Supplementary figure (Fig. S3).

### Ethics and Animal Use Statement

This study was conducted in strict accordance to the recommendations in the Guide for the Care and Use of Laboratory Animals in Japan. Animals were housed at 25 °C with 12-h light/dark cycles and free access to water and standard rodent chow in the Department of Animal Resources of Okayama University. All of procedures and animal protocols was approved by the Committee on the Ethics of Animal Experimentation at Okayama University (Permit No. OKU-2012593). All surgery was performed under general anesthesia with ketamine/pentobarbital, and all efforts were made to minimize animal suffering.

### GBM xenograft and histology

All experiments and protocols were approved by the Institutional Animal Care and Use Committee of Okayama University (OKU-2012593, Japan). Female 9-week-old nude mice (BALB/c-*nu*/*nu*, Japan SLC, Hamamatsu, Japan) were anesthetized by intraperitoneal (*i.p*.) injection of ketamine/pentobarbital and placed in a stereotaxic apparatus. hGICs (10^3^ or 10^4^ cells in 3 μl DMEM) were implanted at the following coordinates: AP, 1.0 mm; ML, 2.5 mm; and DV, 3.0 mm from the bregma. One week after implantation, daily treatment was initiated. Fluvoxamine maleate (50 mg/kg/day, once a day) was injected intraperitoneally every day. In the vehicle control group, mice were administered the same volume (10 ml/kg) of PBS. Four weeks after tumor cell implantation, histological analysis was performed as described in the Supplementary Methods (Doc. S1).

### Statistics

Data are presented as mean ± standard error of the mean (SEM). Data analysis was performed using non-repeated measures analysis of variance (ANOVA) with a Dunnett’s *post-hoc* test. Survival of mice was determined by Kaplan-Meier analysis, and statistical analysis was performed using log-rank test. A statistically significant difference was defined as *p* < 0.05.

## Additional Information

**How to cite this article**: Hayashi, K. *et al*. Fluvoxamine, an anti-depressant, inhibits human glioblastoma invasion by disrupting actin polymerization. *Sci. Rep*. **6**, 23372; doi: 10.1038/srep23372 (2016).

## Supplementary Material

Supplementary Information

## Figures and Tables

**Table 1:** List of drugs used in this study.

No.	Drug	Classification	Manufacturer
1	Chlorpromazine	Typical antipsychotic	Sigma-Aldrich
2	Olanzapine	Atypical antipsychotic	Wako Pure Chemical
3	Quetiapine		Wako Pure Chemical
4	Etizolam	Benzodiazepine	Wako Pure Chemical
5	Flutazolam		Sawai Pharmaceutical
6	Flumazenil	GABA_A_ receptor antagonist	Sigma-Aldrich
7	Imipramine	Tricyclic antidepressant	Nacalai Tesque
8	Clomipramine		Sigma-Aldrich
9	Carpipramine		Mitsubishi Tanabe Pharma
10	Lofepramine		Daiichi Sankyo
11	Trimipramine		Sigma-Aldrich
12	Amitriptyline		Sigma-Aldrich
13	Mianserin	Tetracyclic antidepressant	Sigma-Aldrich
14	Paroxetine	Selective serotonin reuptake inhibitor (SSRI)	Sigma-Aldrich
15	Fluoxetine		Sigma-Aldrich
16	Fluvoxamine		Sigma-Aldrich
17	Sertraline		Sigma-Aldrich
18	Citalopram		Sigma-Aldrich
