# Supplementary material for: Fluvoxamine, an anti-depressant, inhibits human glioblastoma invasion by disrupting actin polymerization
Source: Sci Rep. 2016 Mar 18;6:23372. doi: 10.1038/srep23372 (PMC4796892; doi:10.1038/srep23372)
Supplement: Supplementary Information [file srep23372-s1.pdf]

Title: Fluvoxamine, an anti-depressant, inhibits human glioblastoma invasion by disrupting actin polymerization

Authors: Keiichiro Hayashi[1,2], Hiroyuki Michiue[1*], Hiroshi Yamada[3], Katsuyoshi Takata[4], Hiroki Nakayama[1], Fan-Yan Wei[5], Atsushi Fujimura[1], Hiroshi Tazawa[6,7], Akira Asai[7], Naohisa Ogo[7], Hiroyuki Miyachi[9], Tei-ichi Nishiki[1], Kazuhito Tomizawa[5], Kohji Takei[3] and Hideki Matsui[1]

Supplementary Document S1. (Doc. S1) Supplementary Materials and Methods.

Supplementary Figure 1. (Fig. S1) The inhibition of actin polymerization by fluvoxamine does not involve a direct effect on actin or Arp2/3.

Supplementary Figure 2. (Fig. S2) Fluvoxamine does not affect the phosphorylation levels of ezrin/radixin/moesin, vasodilator-stimulated phosphoprotein, or cofilin.

Supplementary Figure 3. **(Fig. S3)** All of the immunoblotting data in original forms

Supplementary Document S1. (Doc. S1)

Supplementary Materials and Methods

Antibodies.

The following primary antibodies were used in this study: anti-FAK (#3285, Cell Signaling Technology), anti-p-Y397-FAK (#3283, Cell Signaling Technology), anti-p-Y397-FAK (44-624G, Life Technologies), anti-p-Y576/577-FAK (#3281, Cell Signaling Technology), anti-p-Y925-FAK (#3284, Cell Signaling Technology), anti-p-Y118-Paxillin (#2541, Cell Signaling Technology), anti-Src (sc-18, Santa Cruz Biotechnology), anti-p-Y416-Src (#2101, Cell Signaling Technology), anti-p-Y527-Src (#2105, Cell Signaling Technology), anti-Akt (#4691, Cell Signaling Technology), anti-p-S473-Akt (#4060, Cell Signaling Technology), anti-p-T308-Akt (#2965, Cell Signaling Technology), anti-p-S380-PTEN (#9551, Cell Signaling Technology), anti-p-S241-PDK1 (#3438, Cell Signaling Technology), anti-mTOR (#2983, Cell Signaling Technology), anti-p-S2448-mTOR (#2971, Cell Signaling Technology), anti-p-S2481-mTOR (#2974, Cell Signaling Technology), anti-Ezrin/Radixin/Moesin (#3142, Cell Signaling Technology), anti-p-T567-Ezrin/p-T564-Radixin/p-T558-Moesin (#3149, Cell Signaling Technology), anti-VASP (#3132, Cell Signaling Technology), anti-p-S175-VASP (#3111, Cell Signaling Technology), anti-p-S239-VASP (#3114, Cell Signaling Technology), anti-Cofilin (#5175, Cell Signaling Technology), anti-p-S3-Cofilin (#3313, Cell Signaling Technology), anti-Actin (sc-1616-R, Santa Cruz Biotechnology), anti-β-actin (AC-74,

Sigma-Aldrich) and anti-GAPDH (sc-47724, Santa Cruz Biotechnology).

Cell lines and cell culture.

U87-MG, U251-MG and A172 were cultured in DMEM supplemented with 10% FBS, penicillin (100 U/ml) and streptomycin (100 μg/ml). Human glioma-initiating cells (hGICs) were maintained in DMEM/Nutrient F-12 Ham (Sigma-Aldrich) supplemented with N-2 supplement (Life Technologies), L-glutamine (2 mM, Gibco), epidermal growth factor (20 ng/ml, Roche), basic fibroblast growth factor (20 ng/ml, PeproTech), leukemia inhibitory factor (10 ng/ml, Millipore), insulin sodium salt (1 ng/ml, Sigma-Aldrich) and heparin sodium salt (50 μg/ml, Sigma-Aldrich).

Western blotting. Cells grown on Matrigel were washed twice with ice-cold PBS and lysed with ice-cold cell lysis buffer (20 mM Tris-HCl, pH 7.5, 150 mM NaCl, 1 mM EGTA, 1% Triton X-100) containing the Complete protease inhibitor cocktail (Roche) and the PhosSTOP phosphatase inhibitor cocktail (Roche). The lysates were cleared by centrifugation at 15,000 rpm for 30 min at 4°C, separated on SDS-PAGE and transferred to PVDF membrane. The membrane was blocked with 5% BSA for 30 min and incubated with primary antibodies overnight at 4°C. The membrane was incubated with HRP-conjugated appropriate secondary antibodies for 1 h. The immunoreactive bands were visualized by the ECL Prime Western Blotting Detection Reagent (GE Healthcare) and the VersaDoc 5000 MP (Bio-Rad).

Histological analysis.

Mice were deeply anesthetized with pentobarbital and perfused transcardially with PBS, followed by ice-cold 4% PFA. The brains were removed, post-fixed in the same fixative for 24 h at 4°C and embedded in paraffin. Paraffin-embedded sections (4.5-μm thick) were deparaffinized, rehydrated and stained with H&E. Following dehydration and clearing, the sections were mounted with the VectaMount Permanent Mounting Medium (Vector Laboratories).

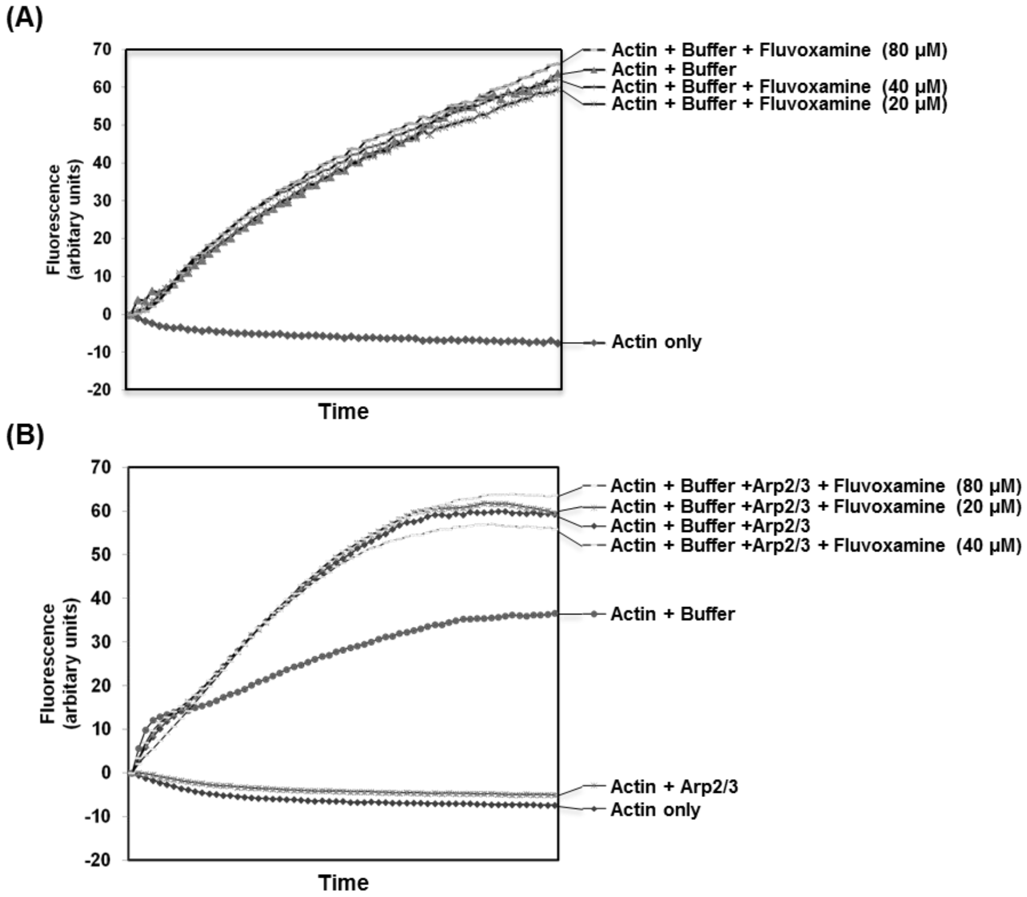

Fig. S1. Inhibition of actin polymerization by fluvoxamine does not involve a direct effect on actin or Arp2/3. (A) Fluvoxamine does not inhibit ion-dependent actin polymerization. Pyrene-actin was polymerized with or without assay buffer. (B) Fluvoxamine does not inhibit Arp2/3-dependent actin polymerization. Pyrene-actin was polymerized with or without purified Arp2/3. Note that these experiments were performed in the absence of mouse brain cytosol.

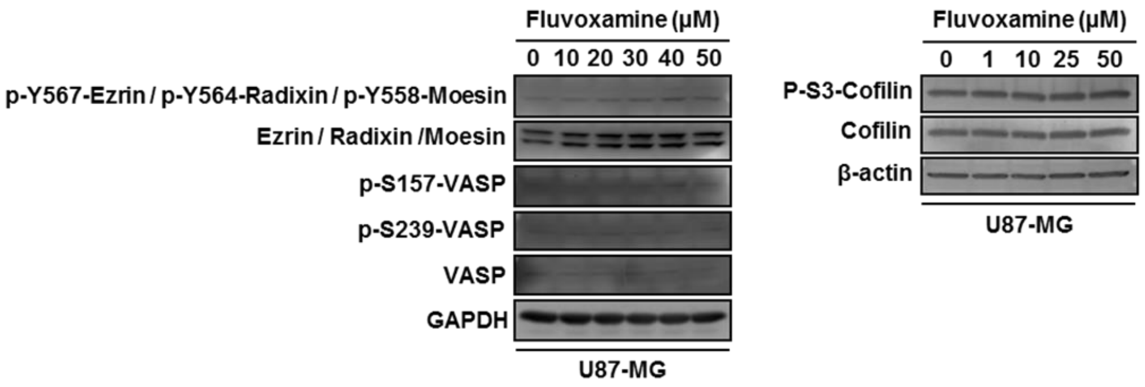

Fig. S2. Fluvoxamine does not affect phosphorylation levels of ezrin/radixin/moesin (ERM) proteins, VASP and cofilin. U87-MG cells were treated with various doses of fluvoxamine for 1h and processed for Western blot analysis.

Fig. S3.

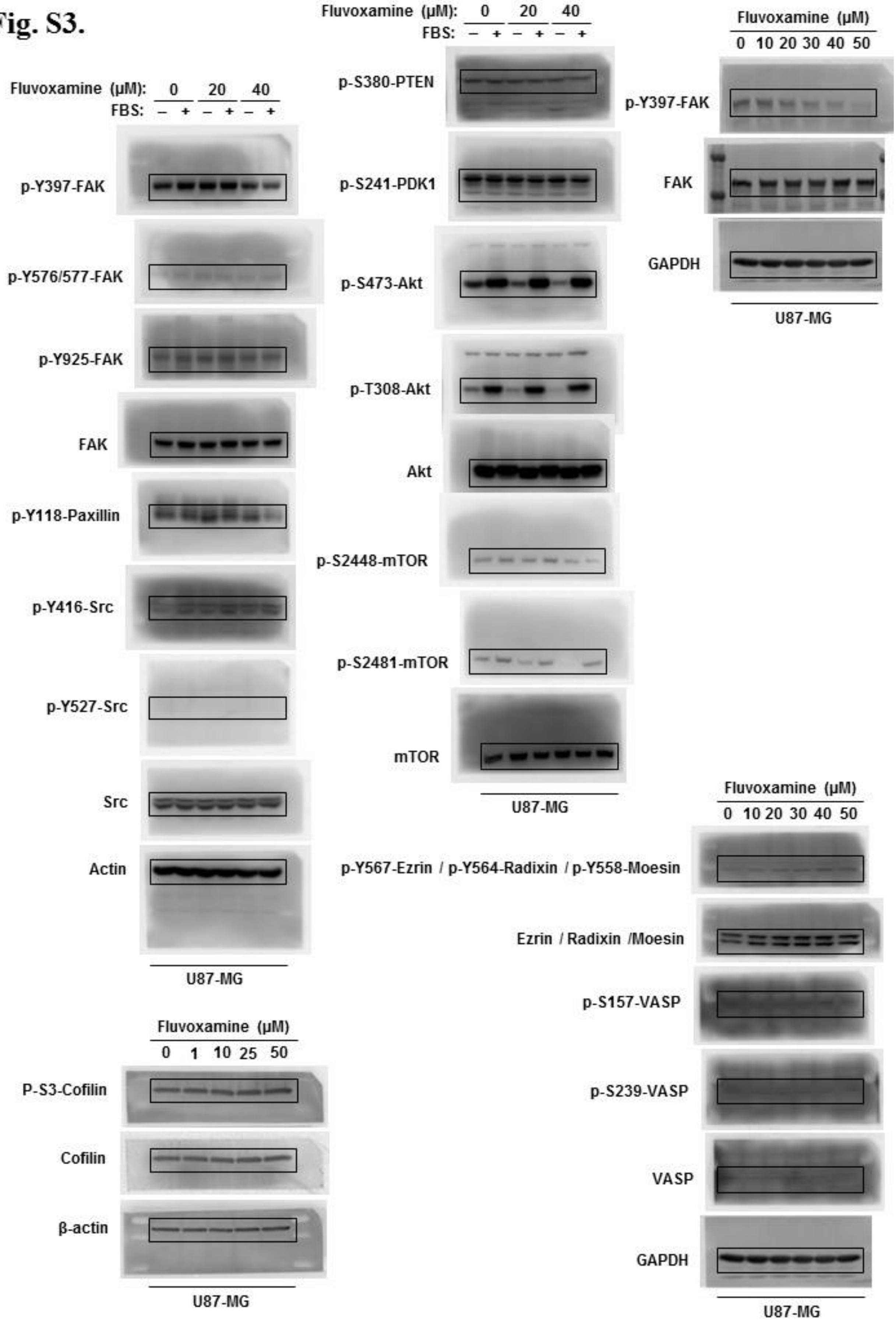
Fluvoxamine (μM): 0 20 40
FBS: – + – + – +
p-Y397-FAK
p-Y576/577-FAK
p-Y925-FAK
FAK
p-Y118-Paxillin
p-Y416-Src
p-Y527-Src
Src
Actin
U87-MG
Fluvoxamine (μM): 0 20 40
FBS: – + – + – +
p-S380-PTEN
p-S241-PDK1
p-S473-Akt
p-T308-Akt
Akt
p-S2448-mTOR
p-S2481-mTOR
mTOR
U87-MG
Fluvoxamine (μM)
0 10 20 30 40 50
p-Y397-FAK
FAK
GAPDH
U87-MG
Fluvoxamine (μM)
0 1 10 25 50
P-S3-Cofilin
Cofilin
β-actin
U87-MG
Fluvoxamine (μM)
0 10 20 30 40 50
p-Y567-Ezrin / p-Y564-Radixin / p-Y558-Moesin
Ezrin / Radixin /Moesin
p-S157-VASP
p-S239-VASP
VASP
GAPDH
U87-MG
